# Identification of Long Non-Coding RNA Profiles and Potential Therapeutic Agents for Fibrolamellar Carcinoma Based on RNA-Sequencing Data

**DOI:** 10.3390/genes14091709

**Published:** 2023-08-28

**Authors:** Janghyun Kim, Young Kim, Bora Lee

**Affiliations:** 1 Department of Oral Pathology, School of Dentistry, Chonnam National University, Gwangju 61186, Republic of Koreayoungkim2017@jnu.ac.kr (Y.K.); 2 Department of Biochemistry, Chonnam National University Medical School, Hwasun 58128, Republic of Korea

**Keywords:** fibrolamellar carcinoma (FLC), long non-coding RNA (lncRNA), therapeutic targets

## Abstract

Background: Fibrolamellar carcinoma (FLC) is a rare type of liver cancer that primarily affects adolescents and young adults without prior liver disease or viral infections. Patients with FLC generally have non-specific symptoms, are often diagnosed at a later stage, and experience a higher frequency of metastases compared to patients with other liver cancers. A fusion transcript of DNAJB1 and PRKACA, which can lead to increased activity of PKA and cellular proliferation, has been identified in all FLC patients, but the exact mechanism through which FLC develops remains unclear. In this study, we investigated common lncRNA profiles in various FLC samples using bioinformatics analyses. Methods: We analyzed differentially expressed (DE) lncRNAs from three RNA sequencing datasets. Using lncRNAs and DE mRNAs, we predicted potential lncRNA target genes and performed Gene Ontology (GO) and KEGG analyses with the DE lncRNA target genes. Moreover, we screened for small-molecule compounds that could act as therapeutic targets for FLC. Results: We identified 308 DE lncRNAs from the RNA sequencing datasets. In addition, we performed a trans-target prediction analysis and identified 454 co-expressed pairs in FLC. The GO analysis showed that the lncRNA-related up-regulated mRNAs were enriched in the regulation of protein kinase C signaling and cAMP catabolic processes, while lncRNA-related down-regulated mRNAs were enriched in steroid, retinol, cholesterol, and xenobiotic metabolic processes. The analysis of small-molecule compounds for FLC treatment identified vitexin, chlorthalidone, triamterene, and amiloride, among other compounds. Conclusions: We identified potential therapeutic targets for FLC, including lncRNA target genes as well as small-molecule compounds that could potentially be used as treatments. Our findings could contribute to furthering our understanding of FLC and providing potential avenues for diagnosis and treatment.

## 1. Introduction

Fibrolamellar carcinoma (FLC) is a rare type of liver cancer that occurs most frequently in adolescents and young adults who have not previously suffered from liver disease or viral infections. It was first described in 1956 by Edmondson, and its unique features were later characterized in the 1980s [1]. Patients with FLC have normal or mildly elevated liver function markers, such as aspartate aminotransferase, alanine aminotransferase, and alkaline phosphatase levels [2], as well as non-specific symptoms, such as abdominal pain, weight loss, and malaise, until a tumor mass is attributed to the symptoms. In addition, multiple studies have reported a significantly higher frequency of metastases and stage IV disease within FLC compared to conventional hepatocellular carcinoma (HCC).

One of the most significant genetic alterations in FLC is a 400-kilobase deletion on chromosome 19. This deletion leads to the fusion of exon 1 in the heat shock protein DNAJB1 with exons 2–10 in PRKACA, the catalytic subunit of protein kinase A [3], thereby promoting tumor growth through the activation of the PRKACA protein kinase [4]. While the rest of the genomic DNA in FLC is relatively unremarkable, the altered expression of various coding and non-coding RNAs has been identified in FLC through a few studies [5,6].

Non-coding RNAs (ncRNAs) are a diverse group of RNAs that do not serve as templates for protein synthesis and have been discovered to occupy a significant portion of the human genome (greater than 90%); they were previously thought to be non-functional and non-coding [7]. Long non-coding RNAs (lncRNAs) are a subset of non-coding RNAs characterized by their extended length, typically longer than 200 nucleotides. They have been implicated in a wide range of cellular processes, including those related to DNA transcription, chromatin modifications, and chromosomal looping, as well as binding to mRNAs and changing their stability, binding to proteins and altering their function, and interacting with other ncRNAs [8,9]. LncRNAs have been shown to play important regulatory roles in gene expression and cellular processes in various diseases. For example, Metastasis-Associated Lung Adenocarcinoma Transcript 1 (MALAT1) promotes the metastasis of lung, breast, pancreas, colon, prostate, and liver cancers by modulating the distribution and levels of active pre-mRNA splicing factors (SR proteins) [10]. Therefore, lncRNAs have been linked to cancer immunoediting, tumor cell and microenvironment (TME) modulation, and immunotherapy resistance. Cancer immunotherapy, which includes Chimeric Antigen Receptor T-Cell Immunotherapy (CART) and immune regulatory checkpoint inhibitors, represents a significant breakthrough in the history of tumor therapy over the last decade. LncRNAs have the capacity to regulate the expression of immune checkpoint genes, such as *programmed cell death 1 (PD-1)*, *programmed cell death ligand 1 (PD-L1)*, *cytotoxic T-lymphocyte-associated protein 4 (CTLA-4)*, and others, either through miRNA sponging or by modulating the NF-kB, PI3K/AKT/mTOR, and WNT pathways [11].

Although a few lncRNAs have been identified in association with FLC progression [5,6], a more comprehensive analysis to uncover a full list of the lncRNAs involved in FLC oncogenesis and progression is needed. Here, we investigated the differentially expressed lncRNA profiles in FLC RNA-seq datasets generated from diverse groups using bioinformatics analyses. We also identified potential lncRNA target genes in these FLC transcriptome profiles. Additionally, we searched for small-molecule compounds to find potential therapeutic targets for FLC. Our results could improve our understanding of FLC and contribute to developing new diagnostic and therapeutic tools.

## 2. Material and Methods

### 2.1. Database Search and Data Acquisition

We searched the Gene Expression Omnibus (GEO) database (https://www.ncbi.nlm.nih.gov/geo/ (accessed on 25 January 2023)) for RNA sequencing datasets related to FLC by using the keywords “fibrolamellar”, “FLC”, and “DNAJ-PKAc”. Three datasets for FLC (GSE63018, GSE73114, and GSE181922) were selected, and the fastq files were downloaded from the European Nucleotide Archive (ENA) using the IBM Aspera tool (v4.0.0.182279). The GSE63018 RNA-seq dataset was published by Sorenson Eric C and includes 9 normal liver samples and 8 FLC liver tumor samples. The GSE73114 RNA-seq dataset was published by Oikawa T and includes 3 adult hepatocyte samples and 4 FLC hepatocellular carcinoma samples. The GSE GSE181922 RNA-seq dataset was published by Francisco Adam B and includes 2 non-malignant liver tissue samples and 7 FLC carcinoma tissue samples. The characteristics of these datasets are provided in Table 1.

### 2.2. RNA-Seq Processing

To remove adapter sequences and improve read quality from three RNA-seq FASTQ files, adapter sequences and low-quality reads were trimmed using Trimmomatic (v0.39) [12]. FastQC was used to check the quality of the trimmed reads and reads with a quality score of less than 20 were discarded. The high-quality trimmed reads were used to analyze lncRNAs and mRNAs. For identifying the lncRNAs, the high-quality reads were aligned to the Gencode v37 human genome file (GRCh38.primary_assembly.genome.fa.gz) using Salmon (v1.4.0) with default settings [13].

### 2.3. Differential Expression Analysis of lncRNA

We evaluated transcript abundance using pseudocounts generated through Salmon quantification, both read numbers and transcripts per million (TPM) counts. We then transformed transcript abundances into gene-level counts using the tximport (v1.6.3) package in R [14]. We annotated the lncRNAs and mRNAs with the Gencode v37 gtf gene annotation files (gencode.v37.long_noncoding_RNAs.gtf.gz and gencode.v37.annotation.gtf.gz). We used the DESeq2 (v1.30.1) package to analyze differential expression between normal and tumor conditions for both lncRNAs and mRNAs [15]. We selected lncRNAs and mRNAs from the three datasets based on the *p*-values calculated by DESeq2. Each RNA sequencing dataset was analyzed independently. The differentially expressed lncRNAs and mRNAs are listed in Supplementary Tables S1 and S2.

### 2.4. Visualization of RNA-Seq Data

The data were standardized and filtered to select significant differentially expressed lncRNAs. A *p*-value less than 0.05 (*p*  <  0.05) and an absolute value of log fold change (FC) greater than 1.5 (|log2FC| > 1.5) were considered to indicate differential expression. Next, the up-regulated and down-regulated differentially expressed lncRNAs in the three datasets were imported into a Venn diagram generator online tool provided by VIB and Ghent University (http://bioinformatics.psb.ugent.be/webtools/Venn/ (accessed on 11 February 2023)). The intersection of the up-regulated and down-regulated differentially expressed lncRNAs in the three datasets was taken. Then, volcano plots and heatmaps of differentially expressed lncRNAs were generated using EnhancedVolcano and ComplexHeatmap (v2.6.2) R packages [16].

### 2.5. Construction of the lncRNA-mRNA Network

To construct a potential lncRNA-mRNA network, we performed Pearson correlation coefficient (PCC) analysis using the Hmisc R package (v4.7.0). The PCC analysis calculated the correlations between 308 lncRNAs and 912 mRNAs. We applied cutoffs of Cor > 0.95 and *p* < 0.01 to identify lncRNAs and mRNAs with significant correlations. The resulting lncRNA–mRNA regulatory network was visualized using Cytoscape, a software platform for visualizing and analyzing biological networks. In Cytoscape, diamond shapes were used to represent lncRNAs, circular (ellipse) shapes were used to represent mRNAs, red nodes were used to represent up-regulated lncRNAs or mRNAs, and blue nodes were used to represent down-regulated lncRNAs or mRNAs. The lncRNA-mRNA regulatory networks are listed in Supplementary Table S3.

### 2.6. Functional Enrichment Analysis

To explore the potential functions of the lncRNA in the FLC samples, we performed Gene Ontology (GO) and Kyoto Encyclopedia of Genes and Genomes (KEGG) pathway analyses using DAVID Bioinformatics Resources (v6.8) (https://david.ncifcrf.gov/ (accessed on 11 February 2023)). GO analysis is frequently used in functional enrichment studies, and KEGG analysis was applied to reveal potential biological pathways associated with differentially expressed genes. Our analysis involved a set of 177 genes that exhibit either a positive or negative correlation with identified lncRNAs in FLC samples. The data visualization was performed using the GOPlot R package. The GO analysis encompassed three categories: biological processes (BP), cellular components (CC), and molecular function (MF). We applied a significance threshold of *p* < 0.05 to determine statistical significance. The functional enrichment results are provided in Supplementary Table S4.

### 2.7. Identification of Small-Molecule Compounds

In our pursuit of uncovering potential therapeutic small-molecule compounds for FLC, we used the Connectivity Map (CMap) database, accessible at http://www.broadinstitute.org/cmap/ (accessed on 11 February 2023). We used the PharmacoGx R package [17,18] to identify promising compounds with therapeutic potential. We focused our inquiry on the top 10 hub genes and used them as queries to interact with the extensive repository of the CMap database. We applied this methodology to extract compounds that exhibited robust connectivity to our gene queries. We selected connectivity scores with a significance level of *p* < 0.05 for further consideration and filtered out those falling outside this range. The identification of potential therapeutic small-molecule compounds is documented in Table 2 and Table S5.

### 2.8. Verification of DE lncRNAs with the TCGA Database

To validate the identified DE lncRNAs, we accessed RNA sequencing data from a cohort of 377 patients diagnosed with hepatocellular carcinoma (HCC). This comprehensive dataset was obtained from the Cancer Genome Atlas (TCGA) database, which is conveniently accessible at https://cancergenome.nih.gov (accessed on 24 April 2023). Firstly, we acquired clinical information of 377 HCC patients by utilizing the data transfer tool of the TCGAbiolinks R package [19]. Secondly, we meticulously carried out a filtration process involving histologic diagnosis and mRNA fusions of specific subtypes, resulting in a precisely curated collection of 5 FLC samples within the broader context of the 377 HCC patient samples. Thirdly, we downloaded and prepared expression profiles for both normal samples (n = 50) and FLC samples (n = 5). Subsequently, all lncRNA expression profiles underwent normalization using the TCGAanalyze_Normalization function of TCGAbiolinks. The normalization of expression profiles involved applying a quantile filter through the TCGAanalyze_Filtering function. The resulting filtered expression profiles were then used to identify the differentially expressed lncRNAs between the normal and FLC samples using the TCGAanalyze_DEA function of TCGAbiolinks. Finally, we annotated all lncRNAs using the Gencode v37 gtf gene annotation file (https://www.gencodegenes.org/ (accessed on 11 February 2023)) (gencode.v37.long_noncoding_RNAs.gtf.gz).

## 3. Results

### 3.1. Identification of Common DE lncRNAs in FLC

**Figure 1:**
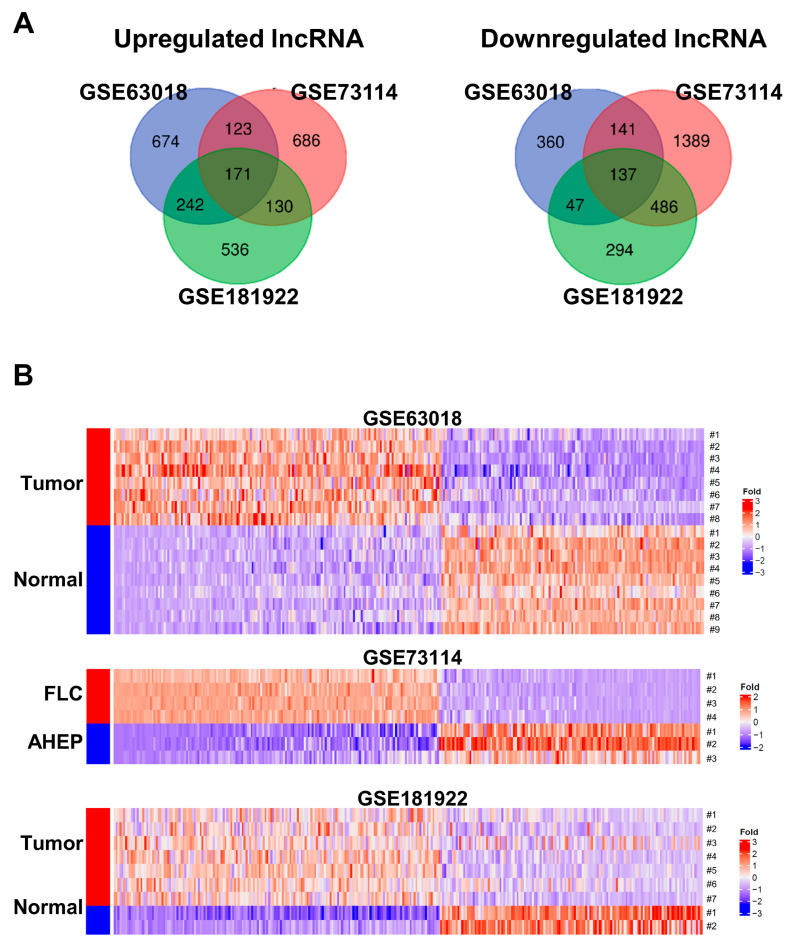

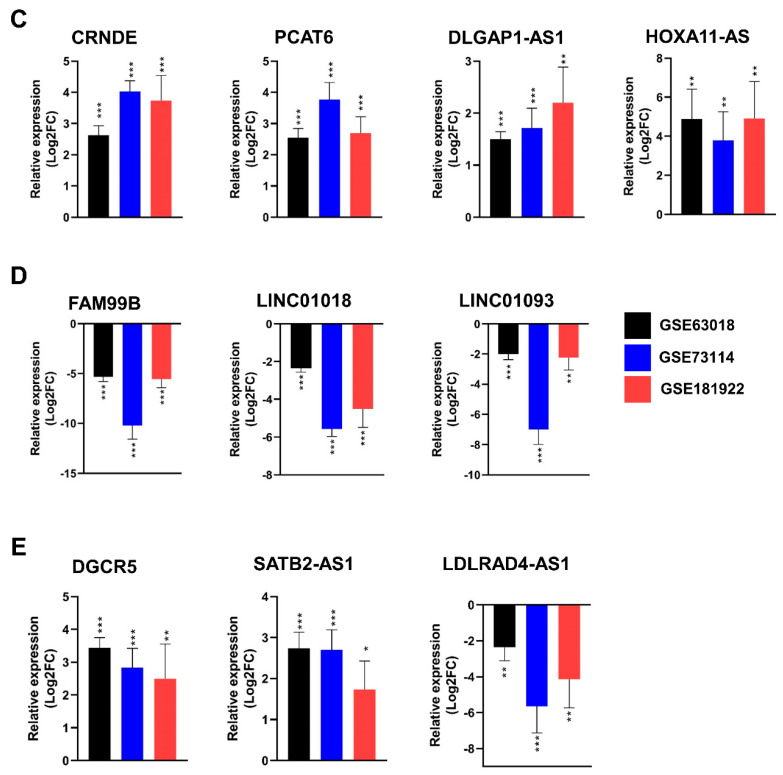
Identification of differentially expressed lncRNA profiles in FLC. The Venn diagram (**A**) and heatmap (**B**) show the number of significantly differentially expressed lncRNA profiles in the FLC samples compared to normal samples. Bar graphs represent the differentially expressed lncRNAs in the three datasets (**C**–**E**). Statistical significance is indicated by asterisks (*: *p* < 0.05, **: *p* < 0.01, ***: *p* < 0.001).

To identify the common differentially expressed lncRNAs in FLC, we selected and analyzed three RNA sequencing datasets from the GEO database (Table 1). From these three datasets, we identified 308 common differentially expressed lncRNAs (of which 171 were up-regulated and 137 were down-regulated) with cutoffs of |log2FC| > 1.5 and values of *p* < 0.05 (Supplementary Table S1). The 308 differentially expressed lncRNAs from the three datasets were visualized using a Venn diagram and heatmap (Figure 1A,B). Several studies have reported that the lncRNAs *colorectal neoplasia differentially expressed* (*CRNDE*), *prostate cancer-associated transcript 6* (*PCAT6*), *DLGAP1 antisense RNA 1* (*DLGAP1-AS1*), and *HOXA11 antisense* (*HOXA11-AS*) are highly expressed in HCC, and their overexpression has been linked to HCC invasion and/or metastasis [20,21,22,23]. In our analysis of the three datasets, we observed that the lncRNAs *CRNDE*, *PCAT6*, *DLGAP1-AS1*, and *HOXA11-AS* were up-regulated in FLC samples (Figure 1C). On the other hand, the lncRNA *family with sequence similarity 99 member B* (*FAM99B*), *long intergenic non-coding RNA 1018* (*LINC01018*), and *long intergenic non-coding RNA 1093* (*LINC01093*) are known to be down-regulated in HCC tissues and cells. In addition, the down-regulation of these lncRNAs has been significantly associated with regulated cell proliferation, cell cycling, cell apoptosis, and poor overall survival in patients with HCC [24,25,26]. Consistent with these findings, we also observed that the lncRNAs *FAM99B*, *LINC01018*, and *LINC01093* were down-regulated in FLC samples (Figure 1D).

Furthermore, the lncRNAs *DiGeorge syndrome critical region gene 5* (*DGCR5*) and *SATB2 antisense RNA 1* (*SATB2-AS1*) are known to be down-regulated in HCC tissues, and their down-regulation has shown a significant correlation with poor overall survival among patients with HCC [27,28]. In contrast, *LDLRAD4 antisense RNA 1* (*LDLRAD4-AS1*) has been reported to exhibit up-regulation in colorectal cancer (CRC) tissue. This up-regulation of *LDLRAD4-AS1* is associated with the promotion of CRC cell migration and invasion in vitro, as well as CRC metastasis in vivo [29]. However, in our study, we observed an opposite trend, where *DGCR5* and *SATB2-AS1* were up-regulated and *LDLRAD4-AS1* was down-regulated in FLC samples (Figure 1E).

### 3.2. Prediction of lncRNA Target Genes

**Figure 2:**
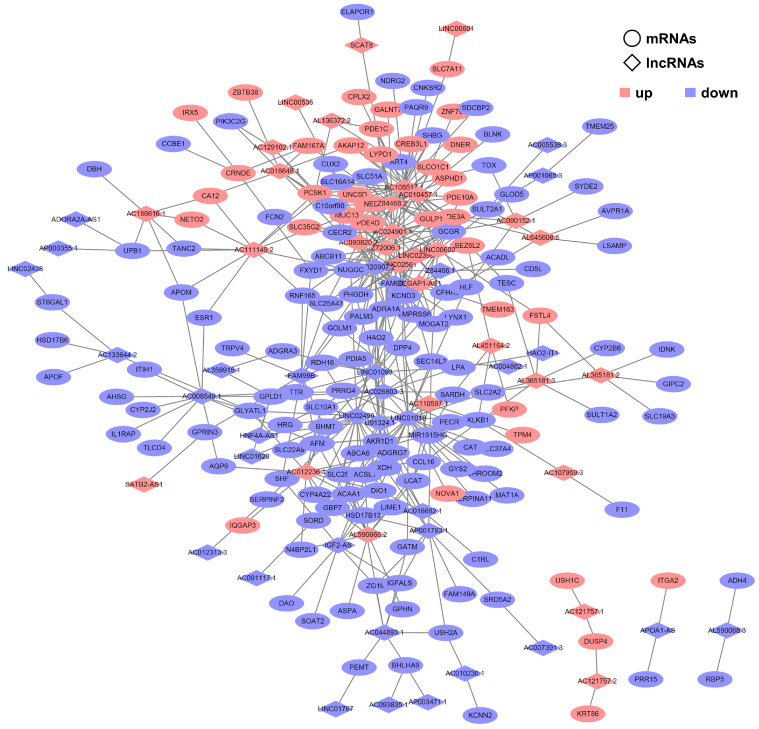
Construction of the FLC-related lncRNA–mRNA network consisting of differentially expressed lncRNAs with their trans-target differentially expressed mRNAs. The diamond and circular shapes represent lncRNAs and mRNAs, respectively. Red nodes represent up-regulated lncRNAs or mRNAs, and blue nodes represent down-regulated lncRNAs or mRNAs.

To explore potential lncRNA target genes in FLC, we conducted a trans-pattern analysis of Pearson correlation coefficients (PCCs) using RNA-seq analysis data, employing the Hmisc package. We specifically focused on PCCs with values satisfying the criteria of *p* < 0.01 and |Cor| > 0.95. We identified 454 common co-expressed pairs with 308 lncRNAs and 912 mRNAs in three RNA-seq datasets. The visual representation of these co-expressed pairs can be observed in Figure 2 using Cytoscape, and the detailed information is accessible in Supplementary Tables S2 and S3. Notably, within this network, the lncRNAs *AC108517.1* and *AC024901.1* exhibited the highest number of interactions, suggesting their potential significance in orchestrating complex molecular relationships. LncRNA *AC108517.1* has been previously reported as being up-regulated in FLC compared to other liver cancers [5]. However, the specific functions and regulatory mechanisms of *AC108517.1* in FLC are unknown, emphasizing the need for further investigation into the precise role of this lncRNA within the FLC landscape.

### 3.3. In Silico Analysis of lncRNA Biomarker Functions

**Figure 3:**
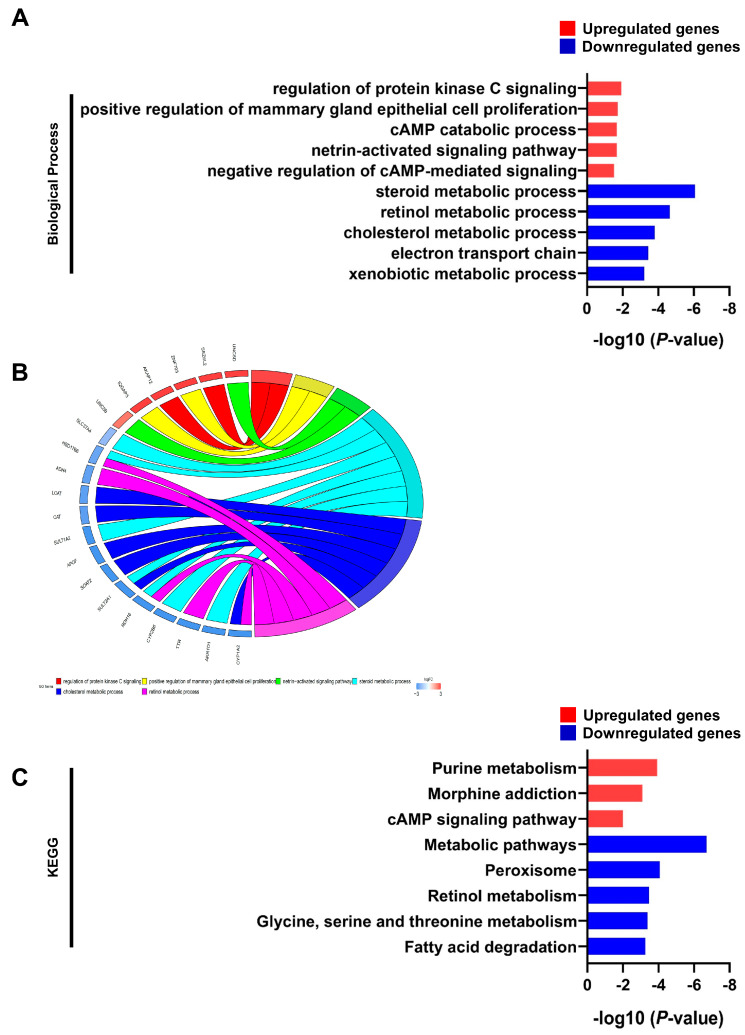
Identification of the biological processes of lncRNA target genes in FLC. Red indicates up-regulation and blue represents down-regulation. The significantly enriched GO terms (**A**) and KEGG pathways (**C**) of both up− and down−regulated target genes are presented. A chord graph illustrates the association between the top three GO items and the hub genes of lncRNA target, with node colors according to the log2FC values (**B**). Red rectangles represent up−regulated target genes and blue rectangle represent down-regulated target genes.

To explore the potential biological roles of target genes of the identified lncRNAs, we performed GO and KEGG function enrichment analyses for the 308 lncRNA-related mRNAs (Supplementary Table S4). In the GO analysis, lncRNA-related up-regulated mRNAs were enriched in the regulation of protein kinase C signaling and cAMP catabolic processes, while lncRNA-related down-regulated mRNAs were enriched in steroid, retinol, cholesterol, and xenobiotic metabolic processes (Figure 3A). The relationship between the hub genes of lncRNA targets and the top three GO biological processes is displayed in Figure 3B. The results of the KEGG analysis showed that lncRNA-related up-regulated mRNAs were enriched in purine metabolism, morphine addiction, and the cAMP signaling pathway, while lncRNA-related down-regulated mRNAs were enriched in metabolic pathways, peroxisomes, retinol metabolism, and fatty acid degradation (Figure 3C).

### 3.4. Identification of Small-Molecule Compounds as Potential Therapeutic Targets for FLC

To identify potential therapeutic targets for FLC in a range of small-molecule compounds, we used the Connectivity Map (CMap) analysis, a well-established approach that explores the intricate relationship between small-molecule compounds and the distinctive gene expression signatures associated with various diseases [17]. We screened for small-molecule compounds based on the differentially expressed hub genes we had identified, using the PharmacoGx R package [18]. We focused on molecular attributes that had the potential to effectively address FLC. From a list of compounds, we selected the top 10 based on their connectivity score and *p*-value (as detailed in Table 2 and Table S5). Among the discerned small-molecule compounds, noteworthy entities such as vitexin, chlorthalidone, triamterene, and amiloride emerged as promising candidates worthy of further investigation and exploration.

### 3.5. Validation of Identified DE lncRNAs in TCGA

**Figure 4:**
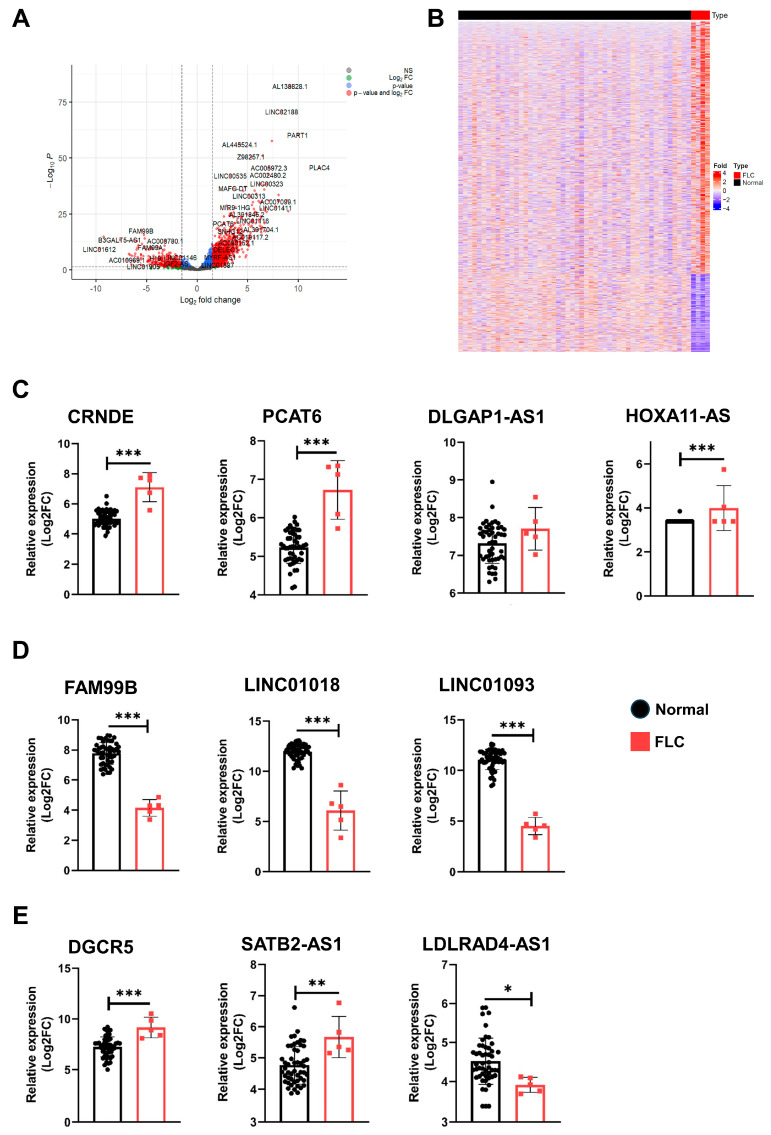
Validation of differentially expressed lncRNAs in TCGA database. The volcano plot (**A**) and heatmap (**B**) show the number of significantly differentially expressed lncRNA profiles in FLC samples compared to normal samples. Bar graphs represent the up- and down-regulated differentially expressed lncRNAs in the TCGA−LIHC dataset (**C**–**E**). Statistical significance is indicated by asterisks (*: *p* < 0.05, **: *p* < 0.01, ***: *p* < 0.001).

To validate the identification of lncRNAs in our study, we utilized the LIHC dataset obtained from The Cancer Genome Atlas (TCGA) database. The TCGA-LIHC dataset, consisting of both normal (n = 50) and FLC samples (n = 5), was analyzed with the TCGAbiolinks tool. Applying cutoffs of |log2FC| > 1.5 and *p* < 0.05, we identified 2100 DE lncRNAs, of which 1351 were up-regulated and 749 were down-regulated (Supplementary Table S6). A volcano plot and heatmap were utilized to visualize these 2100 differentially expressed lncRNAs from the TCGA-LIHC dataset. Interestingly, we observed consistent results in the TCGA-LIHC dataset, with the exception of *DLGAP1-AS1*, as the lncRNAs *CRNDE*, *PCAT6*, and *HOXA11-AS* were all up-regulated. Conversely, we found that the lncRNAs *FAM99B*, *LINC01018*, and *LINC01093* were down-regulated in FLC samples (Figure 4C,D). Furthermore, we observed the up-regulation of *DGCR5* and *SATB2-AS1* and the down-regulation of *LDLRAD4-AS1* in FLC samples (Figure 4E).

## 4. Discussion

Although Dinh and Farber reported a few lncRNAs in FLC [5,6], the exact mechanisms through which lncRNAs are involved in the development of FLC remain largely unknown. To better understand FLC, we conducted a comprehensive analysis of lncRNA expression profiles in multiple FLC samples using bioinformatics techniques. Through this analysis, we identified 308 differentially expressed lncRNAs from three RNA-seq datasets. Multiple studies have reported that the lncRNAs *CRNDE*, *PCAT6*, *DLGAP1-AS1*, and *HOXA11-AS* are highly expressed in HCC and associated with HCC invasion and/or metastasis [20,21,22,23]. Our analysis of three datasets confirmed the up-regulation of these lncRNAs in FLC samples (Figure 1C). On the other hand, the lncRNAs *FAM99B*, *LINC01018*, and *LINC01093* have been found to be down-regulated in HCC tissues and cells, with their down-regulation significantly associated with poor overall survival in HCC patients [24,25,26]. Similarly, our study found that *FAM99B*, *LINC01018*, and *LINC01093* were down-regulated in FLC samples (Figure 1D). These findings suggest that these lncRNAs may have similar roles between HCC and FLC.

Previous studies have reported that the down-regulation of *DGCR5* and *SATB2-AS1*, among the identified lncRNAs, is correlated with poor survival in HCC [27,28]. However, our results demonstrated that *DGCR5* and *SATB2-AS1* were up-regulated in FLC samples. Additionally, the up-regulation of *LDLRAD4-AS1* has been shown to promote CRC cell migration, invasion, and metastasis in vitro and in vivo [29]. Interestingly, our results revealed that *LDLRAD4-AS1* was down-regulated in FLC samples (Figure 1D). These results indicate that FLC possesses unique characteristics that distinguish it from other cancers.

Moreover, the identified lncRNAs *LINC00313*, *LINC00319*, and *MRPL23-AS1* have been found to be up-regulated in testicular germ cell tumors, glioma, and osteosarcoma. Their up-regulation has been significantly correlated with poor overall survival. This correlation has been attributed to their role in modulating the expression of epithelial–mesenchyme transition (EMT)-related proteins, regulating High Mobility Group AT-Hook 2 (HMGA2), and activating Wnt/β-catenin signaling [30,31,32]. We observed that these lncRNAs were significantly up-regulated in FLC samples. These data suggest that they could function similarly in FLC. The possibility that they could serve as prognosis markers in FLC needs further investigation.

To explore the biological functions of the identified lncRNAs, we performed a PCC analysis with differentially expressed lncRNAs and mRNAs. This analysis identified 454 common co-expressed pairs (Figure 2). Among these co-expressed pairs, *AC108517.1* was one of the top hub lncRNAs within the co-expression network. Although lncRNA *AC108517.1* has been reported to be up-regulated in FLC [5], its specific functions and regulatory mechanisms in FLC are still unknown. Our analysis suggests that *AC108515.1* could be one of the master regulators in FLC development and progression and a good target for therapeutic manipulation.

Numerous studies have demonstrated the pivotal role of a specific gene fusion between *DNAJB1* and *PRKACA* in the development of FLC. This gene fusion acts as an oncogenic driver, retaining PKA kinase activity [4]. Furthermore, previous research by Simon has indicated the significant up-regulation of genes associated with cancer-related processes, such as the EGF receptor pathway, cell cycle, glycolysis, and Wnt signaling. Conversely, genes linked to liver functions, particularly retinol/drug metabolism and steroid metabolism, were markedly down-regulated in FLC [33]. We investigated the GO and KEGG pathways of the target genes of the identified lncRNAs and observed that up-regulated target genes of the lncRNAs were associated with protein kinase C signaling and cAMP catabolic processes. Conversely, down-regulated target genes of the lncRNAs were associated with processes involving retinol, cholesterol, and xenobiotic metabolism (Figure 3A,B). These findings consistently align with previous research, reinforcing the notion that the identified lncRNAs potentially hold significant roles in FLC progression. Furthermore, these lncRNAs may serve as regulators, and their manipulation could potentially be explored for FLC treatment strategies.

To identify prospective therapeutic targets for FLC, we performed a CMAP analysis and selected the top 10 small-molecule compounds based on differentially expressed hub genes. Within this selection, the compound vitexin emerges as an intriguing prospect. Previous research has underscored its remarkable capacity to thwart the growth and metastasis of gastric cancer. This pronounced effect has been attributed to the inactivation of the PI3K/AKT/mTOR/HIF-1a signaling cascade [34], accentuating its potential as a potent anticancer agent. Concomitantly, compounds such as chlorthalidone, triamterene, and amiloride emerge, distinguished for their ability to deter sodium reabsorption, a fundamental physiological process. Although the exact functions of these compounds within the context of HCC and FLC are not yet fully understood, their potential as foundational elements for innovative targeted therapies in FLC is highly intriguing.

Finally, we validated the differentially expressed lncRNAs identified in our study utilizing the TCGA database. Specifically, the lncRNAs *CRNDE*, *PCAT6*, and *HOXA11-AS* exhibited an up-regulated trend, while the lncRNAs *FAM99B*, *LINC01018*, and *LINC01093* demonstrated a down-regulated pattern in FLC samples (Figure 4C,D). Additionally, the lncRNAs *DGCR5* and *SATB2-AS1* displayed an up-regulated expression pattern. In contrast, *LDLRAD4-AS1* was significantly down-regulation in FLC samples (Figure 4E). These consistent findings between the commonly identified lncRNAs across the three datasets and those prominently present within the TCGA database underscore the robustness of our study, adding to its credibility and meaningful impact.

## 5. Conclusions

In this study, we used publicly available RNA-seq datasets to explore the lncRNA expression profiles in FLC. Through this comprehensive analysis, we identified shared differentially expressed lncRNAs, pinpointed target genes influenced by these lncRNAs, and uncovered prospective therapeutic targets for FLC. These identified lncRNAs, synergizing with the prospective small-molecule compounds, hold the transformative potential to recalibrate the trajectory of FLC treatment, enhance therapeutic outcomes, and ultimately improve the prognosis for individuals battling this challenging malignancy.

## Figures and Tables

**Table 1:** List of RNA-seq datasets used in the bioinformatics analysis approach.

Accession Number	Platform	Dataset		Reference
		Normal	Tumor	
GSE63018	RNA-seq	GSM1538069, GSM1538074, and GSM1538079 GSM1538081, GSM1538089, and GSM1538091 GSM1538093, GSM1538095, and GSM1538102	GSM1538070, GSM1538073, and GSM1538078 GSM1538080, GSM1538088, and GSM1538092 GSM1538094 and GSM1538101	PMID: 28486549
GSE73114	RNA-seq	GSM1886913, GSM1886914, and GSM1886915	GSM1886919, GSM1886920, GSM1886921, and GSM1886922	PMID: 26437858
GSE181922	RNA-seq	GSM5514457 and GSM5514463	GSM5514456, GSM5514458, and GSM5514459 GSM5514460, GSM5514461, GSM5514462, and GSM5514464	PMID: 35482409

**Table 2:** List of the top 10 most significant small-molecule compounds that could reverse expression of FLC hub genes.

CMAP Name	Connectivity Score	*p*-Value
vitexin	0.72111	0.006397
DipHE Manil metilsulfate	0.70275	0.011382
etodolac	0.66704	0.009131
nadide	0.661135	0.021222
florfenicol	0.640235	0.016044
guanabenz	0.639735	0.025795
cyproterone	0.62461	0.010623
N6-methyladenosine	0.61896	0.021769
acetazolamide	0.60382	0.022488
tocainide	0.59811	0.036806

## Data Availability

The RNA-seq raw data are openly available in the NCBI database (GSE63018, GSE73114, and GSE181922) and in TCGA at https://portal.gdc.cancer.gov/ (accessed on 25 January 2023).

## Supplementary Materials

The following supporting information can be downloaded at: https://www.mdpi.com/article/10.3390/genes14091709/s1, Table S1. List of differentially expressed lncRNAs in each dataset. The log2 fold change values and *p*-values of comparisons between normal and FLC samples were obtained using the Salmon-DESeq2 pipeline. Table S2. List of differentially expressed mRNAs in each dataset. The log2 fold change values and *p*-values of comparisons between normal and FLC samples were obtained using the Salmon-DESeq2 pipeline. Table S3. List of the trans-regulatory genes of differentially expressed lncRNAs. The 81 lncRNAs and their trans-regulatory target mRNAs were identified. Table S4. List of GO terms and KEGG pathway analysis of lncRNA target genes. Table S5. List of potential therapeutic compounds obtained through the CMAP database. Table S6. List of differentially expressed lncRNAs in the TCGA-LIHC dataset. The log2 fold change values and *p*-values of comparisons between normal and FLC samples were obtained using the TCGAbiolinks tool.
